# Exercise-Induced Laryngeal Obstruction: When Pediatric Exertional Dyspnea Does not Respond to Bronchodilators

**DOI:** 10.3389/fped.2019.00052

**Published:** 2019-03-01

**Authors:** J. Tod Olin

**Affiliations:** Department of Pediatrics, National Jewish Health, Denver, CO, United States

**Keywords:** exercise-induced laryngeal obstruction (EILO), exertional dyspnea, continuous laryngoscopy during exercise (CLE), therapeutic laryngoscopy during exercise, Olin EILOBI breathing techniquest

## Abstract

Exertional dyspnea is a common complaint in general pediatric practice. While a high proportion of the general pediatric population has asthma, other diagnoses, including exercise-induced laryngeal obstruction should be considered, especially when asthma therapy is not sufficient to control symptoms. This review describes some of the key clinical features of exercised-induced laryngeal obstruction as well as preferred diagnostic and therapeutic approaches. Importantly, current diagnostic technology has considerably improved in the last decade at specialty centers. At the same time, infrastructure for clinical trials is emerging and there is not strong evidence to support specific therapies at the current time.

## Introduction

Exertional dyspnea is a common presenting complaint in a general pediatric population. It is an important symptom regardless of cause because it may affect quality of life and willingness to exercise in an age characterized by concerns about decreased physical activity (1–3). It also may be a reflection of conditioning status or underlying pathology in a variety of organ systems (4). Finally, treatment of the conditions that cause exercise intolerance may safely increase a child's willingness to exercise, thereby optimizing normal growth and maturation and improving the child's cardiovascular risk profile later in life (5).

Among primary respiratory conditions leading to exertional dyspnea in pediatric populations, asthma is thought to be the most common cause, affecting nearly 300 million people globally and 10% of pediatric populations (6, 7). Exertional symptoms are present in the majority of patients, the impact of other diseases is important for clinicians, researchers, and the general population to consider.

Exercise-induced laryngeal obstruction (EILO) another common condition that causes exertional dyspnea and it is increasingly recognized for its impact on individual patients and populations. The disease is characterized by symptomatic upper airway obstruction at the level of the glottis or supraglottis during exercise in the absence of symptoms or obstruction at rest (8). In the evaluation of exertional dyspnea, EILO is a condition that warrants attention alongside asthma in selected populations, but our understanding of the condition is primitive when compared to asthma. This review will summarize this understanding of EILO and outline potential areas of high-yield scientific growth in the future.

## Definition, Nomenclature, and Description

In 2015, an international expert panel published a consensus document which discussed definitions and nomenclature related to episodic shortness of breath caused by the upper airway (8). “Inducible laryngeal obstruction” was introduced as the preferred term to describe glottic or supraglottic obstruction which occurred in response to a variety of environmental, psychologic, or exertional triggers. EILO is the term used to describe this phenomenon when the trigger is isolated to exercise. It replaces previously used terms including vocal cord dysfunction and paradoxical vocal fold motion.

EILO is defined by the presence of laryngoscopically visible upper airway obstruction that occurs during exercise before self-resolving (8). By definition, this same obstruction does not occur at rest. Historically, many of the descriptions of this condition highlighted involvement of the vocal folds. EILO can also be caused by a variety of structures in the supraglottic region including the arytenoid cartilages, tissue in the intra-arytenoid space, aryepiglottic folds, and rarely, the epiglottis. Although obstruction at either the glottic or supraglottic level is sufficient to make a diagnosis, the precise degree of obstruction at either anatomic level which differentiates 1) normal upper airway function from 2) an observed abnormality lacking clinical significance from 3) a clinical problem has not been defined.

## Epidemiology and Impact

The precise incidence and prevalence of this condition across a cross section of a population representing a variety of races and ethnicities has not been perfectly defined. Studies published to assess EILO prevalence have varied in terms of diagnostic methodologies. Nonetheless, in northern Europe, two studies both estimated that the population prevalence of EILO across adolescents and young adult seems to be in the range of 5% (9, 10).

Other studies have assessed sample EILO prevalence in referral clinics as well as patient characteristics among samples of patients previously diagnosed with EILO. Within clinics focused on refractory asthma or exertional dyspnea, EILO has been detected in a large, but highly variable proportion of patients (11–14). Many of these studies as well as others document and unequal gender distribution of identified subjects with a strong female predominance (15, 16). When race and ethnicity is reported, there is a notable Caucasian predominance (15, 16). There is not a clear understanding of changes in disease prevalence across the age spectrum.

## Pathophysiology

The mechanism of EILO is not completely understood. A variety of intrinsic and extrinsic contributors may be important. Anatomic factors, including airway size or airway pliability, logically impact the physics of airflow and may contribute to the frequency and severity of disease (17, 18).

Upper airway microanatomic factors could impact stimuli processing or the magnitude of inappropriate neuromuscular responses, possibly mediated through epithelial, neurologic, or muscular dysfunction (19).

The literature has highlighted a behavioral phenotype of EILO patients (20). Our understanding of the relevance of these behavioral observations is primitive and causality is certainly not proven.

Potential extrinsic contributors to the process of inappropriate upper airway obstruction during exercise include asthma, reflux, and nasal disease (21). Asthma triggers and EILO triggers are not necessarily identical (22). In EILO, upper airway abnormalities have been detected almost exclusively during high-intensity exercise (15). Surrogate testing has not yet been identified.

## Clinical presentation

Many of the case reports and case series focusing on EILO discuss competitive young females presenting with frightening inspiratory stridor during strenuous exercise which resolves within minutes of exercise termination. Sometimes a personality phenotype characterized by perfectionistic or anxiety spectrum features co-travels with the description of symptoms (23).

In clinical practice, disease variability is likely extensive. The presence or absence of stridor may simply depend on disease severity and patient size (which is an important determinant of airflow) and extensive focus on stridor has the potential to mislead clinicians into discarding the diagnosis in smaller patients. Patient demographic features including age, gender, and athletic level have varied in the literature. A 32-year-old female presenting with long-standing exertional stridor secondary to supraglottic obstruction was one of the first cases presented in the literature (24). Young children with prominent supraglottic obstruction may also present with stridor, especially those known to have a degree of congenital laryngomalacia (18). The literature describes a female predominance, yet at the same time, males make up a large proportion of patients. Some cases come to light in the evaluation of “steroid-resistant asthma” (25, 26).

At an anecdotal level, our experience at a referral center that focuses on exertional dyspnea supports messages of EILO variability and the importance of curiosity during clinical histories.

We have recognized age as potentially misleading. Early school-age patients, later found to have prominent supraglottic EILO via continuous laryngoscopy during exercise (CLE), have initially presented with highly consistent exertional stridor which is not frightening to the patient, but highly concerning to observers. Patients in their 50s, later found to have prominent glottic EILO, have presented with ”steroid-dependent asthma“ and significant side effects from chronic oral steroid use including weight increases of over 50 kg over the course of therapy.

We are concerned that excessive focus on athletic level has the potential to unconsciously affect the rigor of diagnostic evaluation was performed by clinicians. In this line of thinking, we are concerned that patients not performing at a high level will be less likely to receive extensive exercise diagnostics because the perceived impact of exertional symptoms seems lower.

We have recognized descriptions of symptoms across the respiratory cycle as potentially misleading. Patients of almost all ages frequently refer to all audible respiratory noise as “wheeze,” even those later to proven to struggle with isolated inspiratory obstruction with EILO. Many patients with severe obstructive disease, later proven to be unaffected by EILO, seem to describe symptoms as inspiratory despite the physiology of their diseases.

While not published, we have recognized excessive focus on audible stridor to be potentially misleading as well. Smaller patients may not have airflows sufficient to generate an audible noise or findings may be subtle (Video 1). Other patients, despite reproduction of obvious audible stridor during exercise testing, do not seem to be aware of the noise and subsequently do not report it to healthcare providers.

## Diagnostic Evaluation With Consideration of Differential Diagnosis

The diagnostic evaluation of EILO should be one part of a general evaluation looking and causes of exertional dyspnea. In the pediatric population, as part of this evaluation, it is reasonable to consider both very common and potentially dangerous conditions.

**Figure 1:**
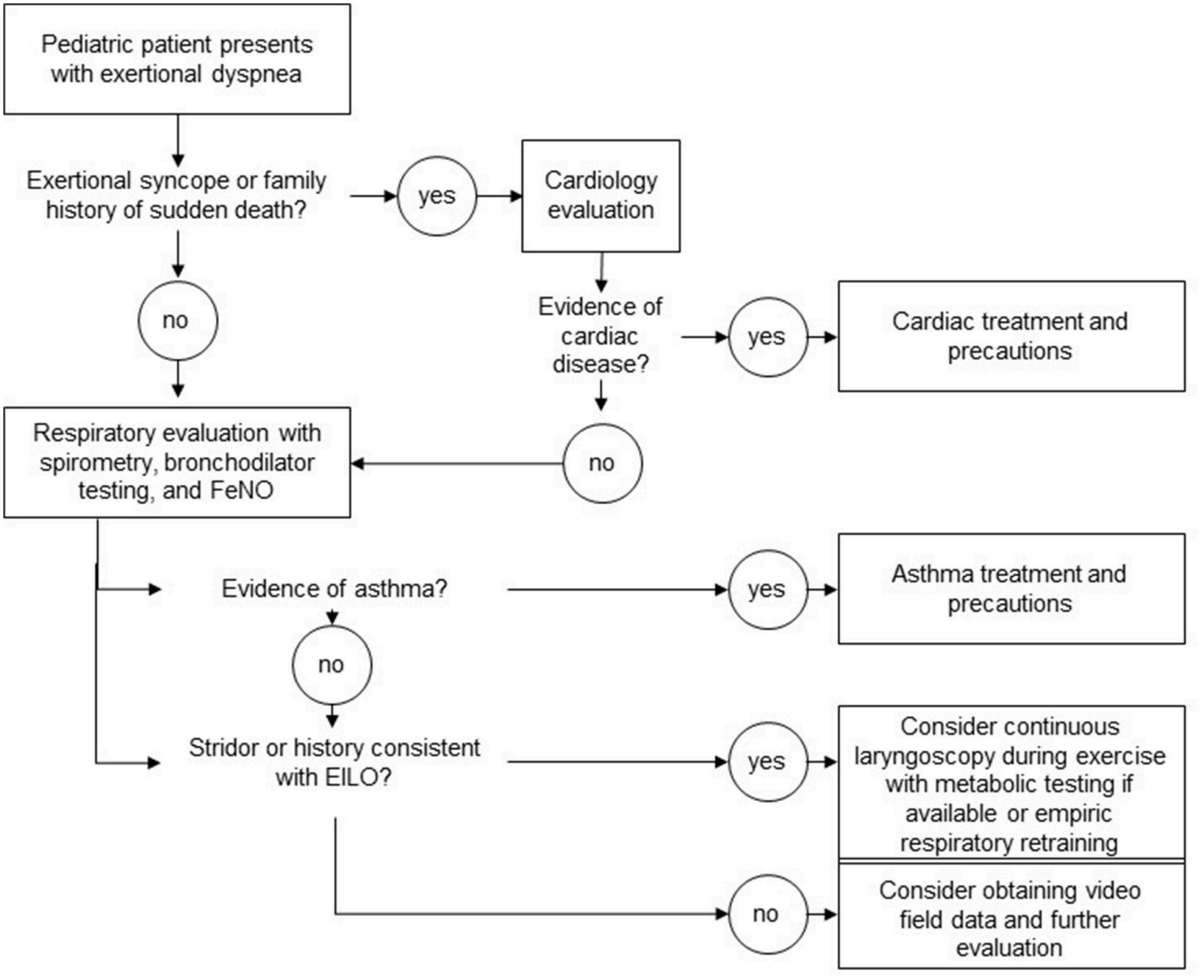
A suggested diagnostic algorithm for young athletes complaining of exertional dyspnea.

When considering respiratory causes of exertional dyspnea, clearly asthma needs to be considered (Figure 1). In pediatric populations, many patients with exercised-induced bronchoconstriction struggle with non-exertional symptoms of cough and wheeze at baseline or during viral illness. As asthma is quite common, we recommend the use of spirometry and bronchodilator testing as an initial screen. At a more detailed level, and depending on available resources, provocative testing with methacholine challenge or eucapnic voluntary hyperventilation can be considered.

Cardiac causes of exertional dyspnea within pediatric populations, in whom congenital heart disease was presumably identified in infancy, are not as common as respiratory causes (given that asthma affects roughly 10% of the population). Importantly, identification of audible adventitial sounds should direct the clinical evaluation away from extensive cardiac testing because adventitial sounds are confined to the airway space. Syncope is a symptom that should always trigger a rigorous cardiac evaluation as left-sided obstructive lesions and dysrhythmias can be fatal.

Presyncope is a challenging coexisting complaint to evaluate. It can be associated with a number of cardiac, autonomic, and respiratory conditions. When clearly linked temporally with coexisting respiratory symptoms, such as described hyperventilation, it may be reasonable to defer extensive cardiac evaluation.

Once attention is turned to EILO, clinicians may be burdened by the relatively large number of methods described in the literature to evaluate the condition. Published case series have included subjects through the use of clinical history alone, flow volume loop analysis at rest, flow volume loop analysis during exercise, auscultation during exercise, ultrasound-based approaches, impulse oscillometry, resting laryngoscopy, post-exercise laryngoscopy, pre-and post-exercise laryngoscopy, and CLE (27–31).

## Continuous Laryngoscopy During Exercise

**Figure 2:**
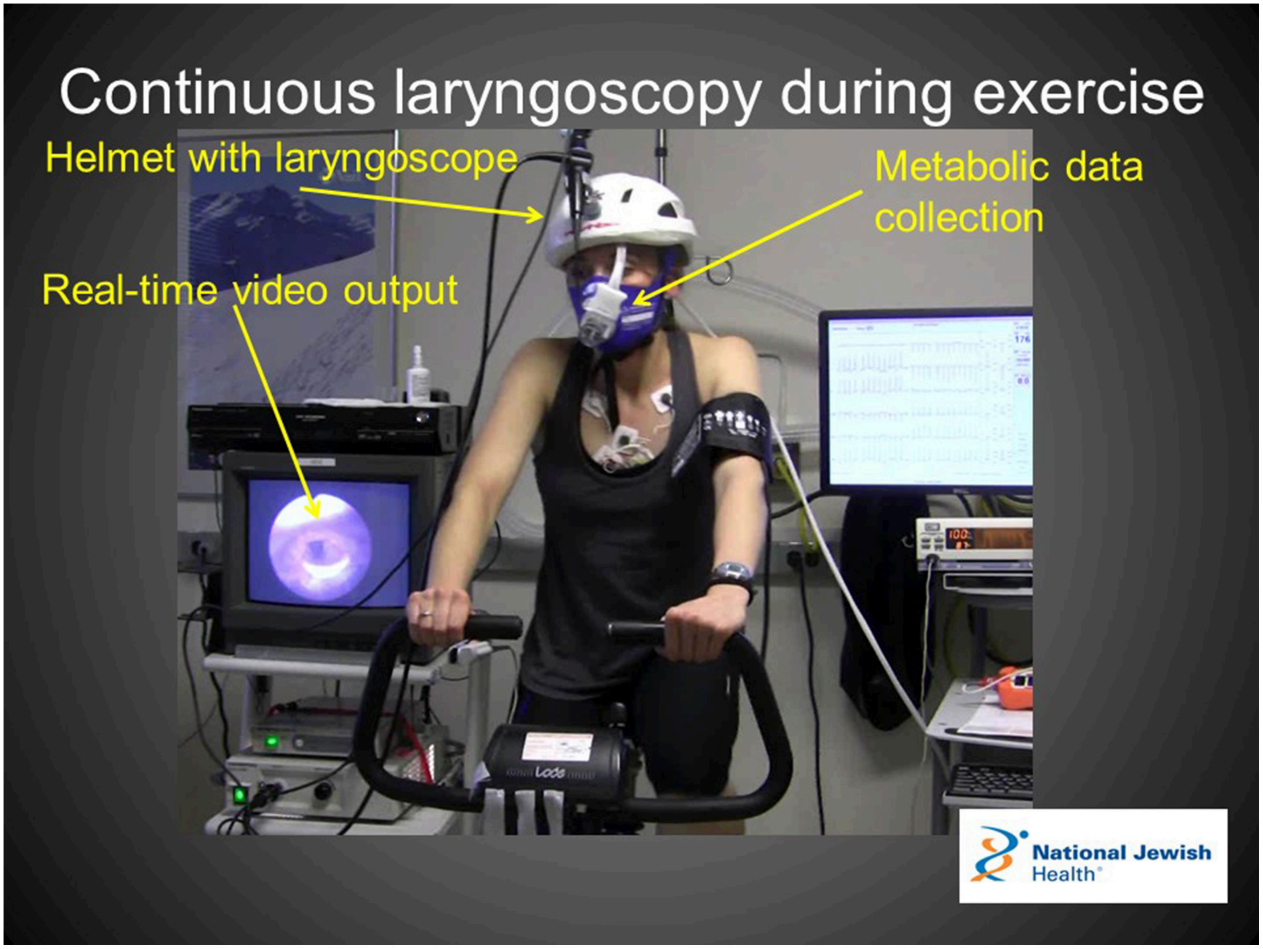
Continuous laryngoscopy during exercise apparatus enables real-time visualization of the larynx with simultaneous ventilator and metabolic data collection. Verbal and written consent for the use of this image was obtained from the patient for use in this manuscript.

In centers with appropriate resources to perform safe and tolerable examinations, we recommend CLE as a preferred diagnostic approach over resting laryngoscopy and post-exertional laryngoscopy. In this procedure, a flexible laryngoscope is introduced prior to exercise and remains in place throughout an exercise challenge intended to reproduce characteristic field symptoms. CLE is reported in the literature as part of exercise testing in a variety of exercise modes including treadmill ergometry, cycle ergometry, rowing, and swimming (31–34). Each exercise modality presents unique challenges to proceduralists, with cycle ergometry likely being the safest and most feasible of the above exercise modes (Figure 2). Centers interested in pursuing this diagnostic approach also need to consider the relative likelihood of reproducing field symptoms across these different exercise modes in any given patient. Some authors have advocated for the use of treadmill ergometry as the preferred exercise mode for this reason (35).

There are multiple lines of reasoning that support the use of CLE as the preferred diagnostic method for EILO when available resources support the possibility. Importantly, the literature describes the visualized endoscopic findings of EILO patients as a function of exercise intensity (15). This study clearly describes an absence of resting findings as well as an absence of visualized findings during moderate exercise intensity. This study also describes a very rapid resolution of visualized findings upon exercise termination. Secondly, laryngoscopy can occasionally be an uncomfortable procedure if not performed by an experienced endoscopist. Performing a laryngoscopy after intense exercise during a period of shortness of breath has the potential to introduce abnormal laryngeal movements that do not accurately represent glottic behavior during intense exercise. Finally, although not quantitatively described in published literature, in our experience patients demonstrate an improved level of diagnostic acceptance and understanding after observing endoscopic and external video footage of observe symptoms which are perceived to reproduce characteristic field symptoms.

## Therapeutic Options

### Medical Therapies

Currently, EILO therapies likely vary across centers and regions. Studies have documented case-based successes of medical, surgical, and behavioral therapies. There have not been published prospective randomized trials with well-described therapies on patients with symptoms isolated to exercise. There is a case series of patients with non-specific ILO in which tricyclic antidepressants were concluded to demonstrate potential benefit (36). This therapy is not recommended at the current time for EILO because patients with symptoms isolated to exercise were not a focus of this trial. The use of inhaled anticholinergic agents has also been proposed (37). Anticholinergic agents theoretically decrease muscarinic stimulus of laryngeal adductors and may favorably alter upper airway secretions.

Medical therapy may be more relevant for the treatment of conditions which may contribute to the presentation of EILO. Asthma, nasal disease, gastroesophageal reflux, and behavioral health disorders may theoretically affect EILO and may be amenable to medical therapy.

### Surgical Therapies

Surgery may be a reasonable option for patients with EILO in patients with a prominent supraglottic component to the disease. Some European laryngology groups have used supraglottoplasty for selected patients because some of the laryngeal findings are reminiscent of patients with congenital laryngomalacia (38–42). The specific surgical approach includes release of the aryepiglottic folds and reduction in the size of the arytenoid towers. While initial reports of the approach seemed promising, it is important to understand that there is not a published body of data describing complications of the procedure.

Botulinum toxin, which can be injected into specific structures in the larynx, has been proposed as a potential therapy for patients that fail non-invasive therapies (43–45). As with medical therapies noted above, there is not sufficient evidence in patients with symptoms limited to exercise to warrant a recommendation for the use of this therapy.

### Behavioral Therapies

Speech-language pathology intervention has been noted to be a potential therapy since the early reports of irritant-associated ILO were published (46). Specific breathing techniques taught by speech and language pathologists may differ across centers (47–50). General relaxation and biofeedback have also been incorporated in some of the therapies (16, 51). Behavioral therapies do not carry specific medical or surgical risks, but the potential positive effects of these therapies are challenging to study as they are likely multifactorial.

## Future

There are a number of very important gaps in our understanding of EILO. These range from basic science considerations to detailed treatment considerations.

Most importantly, the subtypes and associated mechanisms of EILO are very poorly understood. The impact of this problem is obvious when one considers the very broad spectrum of treatment approaches for the disease which may vary across regions of the world. As noted above, there are published schools of thought that focus on medical interventions with anticholinergic agents or reflux suppression, surgical interventions with supraglottoplasty, and behavioral health interventions with respiratory retraining, biofeedback, and psychotherapy.

Working backwards from these empiric therapies, is possible to hypothesize that the primary physiologic abnormality underlying the condition varies across different subtypes of disease yet to be identified definitively. It is also possible to hypothesize that the mechanism of disease relates to structural considerations of airway size and macro anatomy, epithelial integrity and micro-anatomy, airway muscles, airway nerves, intrinsic or extrinsic airway irritants, or central nervous processes.

At the current time, the hurdles to elucidating disease mechanism are numerous. They include the lack of a definitive categorization system of different disease subtypes and the lack of a widely-agreed-upon metric of disease severity. There is also a lack of feasible assays to assess airway size, airway pliability, airway irritability, epithelial integrity, muscle function, nerve function, and instantaneous central nervous stress.

In addition to a developing understanding of disease mechanism, there is a poor understanding of the differences observed across patients in terms of clinical presentation. For example, why do patients with similar degrees of airway obstruction observed on CLE present with seemingly different histories in terms of symptom quantity, sensation perception, and disease impact?

There are important gaps to fill in terms of diagnostic testing. While recent literature suggests that CLE is the preferred methodology for diagnosing the condition. There are still many unanswered question regarding exercise mode, exercise protocol, and environmental conditions, the answers to which will impact test sensitivity, specificity, and predictive value.

Finally, there are many unanswered questions related to disease treatment. It is possible to hypothesize that different schools of thought regarding disease treatment evolved across different medical specialties. As noted above, seminal literature on the condition developed within the fields of otolaryngology and pulmonology. Subsequently, authors from a number of different specialties including general medicine, allergy/immunology, sports medicine, and performance psychology have contributed to our collective knowledge of disease treatment. Ultimately, a better understanding of disease mechanism will lead to more personalized treatments.

In terms of quantifying the effects of these treatments and comparing them against one another, improved clinical trials infrastructure is required. Both physiologic in patient-reported measures of short-term and long-term disease impact will be required. Clinical trials will also rely on standardization of provocative protocols. Differences reported across centers will need to be understood as well.

## Author Contributions

The author confirms being the sole contributor of this work and has approved it for publication.

### Conflict of Interest Statement

The author declares that the research was conducted in the absence of any commercial or financial relationships that could be construed as a potential conflict of interest.

## Supplementary Material

Video 1 Field videos, such as this one demonstrating mild inspiratory stridor, can be a helpful diagnostic tool for clinicians. Verbal and written consent for the use of this video was obtained from the patient for use in this manuscript. Reproduced with permission from National Jewish Health.

## References

[1] Hallstrand TS Curtis JR Aitken ML Sullivan SD. Quality of life in adolescents with mild asthma. Pediatr Pulmonol. (2003) 36:536–43. 10.1002/ppul.10395 14618647 PMC2002508

[2] Glazebrook C McPherson AC Macdonald IA Swift JA Ramsay C Newbould R. Asthma as a barrier to children's physical activity: implications for body mass index and mental health. Pediatrics. (2006) 118:2443–9. 10.1542/peds.2006-1846 17142530

[3] Dantas FM Correia MA Jr Silva AR Peixoto DM Sarinho ES Rizzo JA. Mothers impose physical activity restrictions on their asthmatic children and adolescents: an analytical cross-sectional study. BMC Public Health. (2014) 14:287. 10.1186/1471-2458-14-287 24673939 PMC4230244

[4] Nadeau KJ Zeitler PS Bauer TA Brown MS Dorosz JL Draznin B. Insulin resistance in adolescents with type 2 diabetes is associated with impaired exercise capacity. J Clin Endocrinol Metab. (2009) 94:3687–95. 10.1210/jc.2008-2844 19584191 PMC2758737

[5] Kavey RE Daniels SR Lauer RM Atkins DL Hayman LL Taubert K. American heart association guidelines for primary prevention of atherosclerotic cardiovascular disease beginning in childhood. J Pediatr. (2003) 142:368–72. 10.1067/mpd.2003.205 12712052

[6] Masoli M Fabian D Holt S Beasley R Global Initiative for Asthma P. The global burden of asthma: executive summary of the GINA Dissemination Committee report. Allergy. (2004) 59:469–78. 10.1111/j.1398-9995.2004.00526.x 15080825

[7] Akinbami LJ Moorman JE Bailey C Zahran HS King M Johnson CA Trends in asthma prevalence, health care use, and mortality in the United States, 2001–2010. NCHS Data Brief. (2012) 94:1–8. 22617340

[8] Christensen PM Heimdal JH Christopher KL Bucca C Cantarella G Friedrich G. ERS/ELS/ACCP 2013 international consensus conference nomenclature on inducible laryngeal obstructions. Eur Respir Rev. (2015) 24:445–50. 10.1183/16000617.00006513 26324806 PMC9487687

[9] Christensen PM Thomsen SF Rasmussen N Backer V. Exercise-induced laryngeal obstructions: prevalence and symptoms in the general public. Eur Arch Otorhinolaryngol. (2011) 268:1313–9. 10.1007/s00405-011-1612-0 21528411

[10] Johansson H Norlander K Berglund L Janson C Malinovschi A Nordvall L. Prevalence of exercise-induced bronchoconstriction and exercise-induced laryngeal obstruction in a general adolescent population. Thorax. (2015) 70:57–63. 10.1136/thoraxjnl-2014-205738 25380758

[11] Hira HS Singh A. Significance of upper airway influence among patients of vocal cord dysfunction for its diagnosis: Role of impulse oscillometry. Lung India. (2009) 26:5–8. 10.4103/0970-2113.45197 20165587 PMC2813109

[12] Roksund OD Maat RC Heimdal JH Olofsson J Skadberg BT Halvorsen T. Exercise induced dyspnea in the young. Larynx as the bottleneck of the airways. Respir Med. (2009) 103:1911–8. 10.1016/j.rmed.2009.05.024 19782550

[13] Nielsen EW Hull JH Backer V. High prevalence of exercise-induced laryngeal obstruction in athletes. Med Sci Sports Exerc. (2013) 45:2030–5. 10.1249/MSS.0b013e318298b19a 23657163

[14] Tilles SA Ayars AG Picciano JF Altman K. Exercise-induced vocal cord dysfunction and exercise-induced laryngomalacia in children and adolescents: the same clinical syndrome? Ann Allergy Asthma Immunol. (2013) 111:342–346 e341. 10.1016/j.anai.2013.07.025 24125138

[15] Olin JT Clary MS Fan EM Johnston KL State CM Strand M. Continuous laryngoscopy quantitates laryngeal behaviour in exercise and recovery. Eur Respir J. (2016) 48:1192–200. 10.1183/13993003.00160-2016 27418554

[16] Olin JT Deardorff EH Fan EM Johnston KL Keever VL Moore CM. Therapeutic laryngoscopy during exercise: a novel non-surgical therapy for refractory EILO. Pediatr Pulmonol. (2017) 52:813–9. 10.1002/ppul.23634 27797458

[17] Wysocki J Kielska E Orszulak P Reymond J. Measurements of pre- and postpubertal human larynx: a cadaver study. Surg Radiol Anat. (2008) 30:191–9. 10.1007/s00276-008-0307-8 18253693

[18] Hilland M Røksund OD Sandvik L Haaland Ø Aarstad HJ Halvorsen T. Congenital laryngomalacia is related to exercise-induced laryngeal obstruction in adolescence. Arch Dis Child. (2016) 101:443–8. 10.1136/archdischild-2015-308450 26906070 PMC4853585

[19] Morrison M Rammage L Emami AJ. The irritable larynx syndrome. J Voice. (1999) 13:447–55. 10.1016/S0892-1997(99)80049-6 10498060

[20] Husein OF Husein TN Gardner R Chiang T Larson DG Obert K. Formal psychological testing in patients with paradoxical vocal fold dysfunction. Laryngoscope. (2008) 118:740–7. 10.1097/MLG.0b013e31815ed13a 18182970

[21] Phua SY McGarvey L Ngu M Ing A. The differential effect of gastroesophageal reflux disease on mechanostimulation and chemostimulation of the laryngopharynx. Chest. (2010) 138:1180–5. 10.1378/chest.09-2387 20558553

[22] Walsted ES Hull JH Sverrild A Porsbjerg C Backer V Bronchial provocation testing does not detect exercise-induced laryngeal obstruction. J Asthma. (2017) 54:77–83. 10.1080/02770903.2016.1195843 27285291

[23] McFadden ER Jr Zawadski DK. Vocal cord dysfunction masquerading as exercise-induced asthma. a physiologic cause for “choking” during athletic activities. Am J Respir Crit Care Med. (1996) 153:942–7. 10.1164/ajrccm.153.3.8630577 8630577

[24] Lakin RC Metzger WJ Haughey BH. Upper airway obstruction presenting as exercise-induced asthma. Chest. (1984) 86:499–501. 10.1378/chest.86.3.499 6468016

[25] Newman KB Mason UG III Schmaling KB. Clinical features of vocal cord dysfunction. Am J Respir Crit Care Med. (1995) 152(4 Pt 1):1382–6. 10.1164/ajrccm.152.4.7551399 7551399

[26] Thomas PS Geddes DM Barnes PJ. Pseudo-steroid resistant asthma. Thorax. (1999) 54:352–6. 10.1136/thx.54.4.352 10092698 PMC1745468

[27] De Guzman V Ballif CL Maurer R Hartnick CJ Raol N. Validation of the dyspnea index in adolescents with exercise-induced paradoxical vocal fold motion. JAMA Otolaryngol Head Neck Surg. (2014) 140:823–8. 10.1001/jamaoto.2014.1405 25104182

[28] McNally P Greally P. Use of post-exercise laryngoscopy to evaluate exercise induced dyspnea. Pediatric Pulmonol. (2010) 45:1037–9. 10.1002/ppul.21280 20717906

[29] Chiang T Marcinow AM deSilva BW Ence BN Lindsey SE Forrest LA. Exercise-induced paradoxical vocal fold motion disorder: diagnosis and management. Laryngoscope. (2013) 123:727–31. 10.1002/lary.23654 23097011

[30] Komarow HD Young M Nelson C Metcalfe DD. Vocal cord dysfunction as demonstrated by impulse oscillometry. J Allergy Clin Immunol Pract. (2013) 1:387–93. 10.1016/j.jaip.2013.05.005 24565544 PMC4830384

[31] Heimdal JH Roksund OD Halvorsen T Skadberg BT Olofsson J. Continuous laryngoscopy exercise test: a method for visualizing laryngeal dysfunction during exercise. Laryngoscope. (2006) 116:52–7. 10.1097/01.mlg.0000184528.16229.ba 16481809

[32] Tervonen H Niskanen MM Sovijarvi AR Hakulinen AS Vilkman EA Aaltonen LM. Fiberoptic videolaryngoscopy during bicycle ergometry: a diagnostic tool for exercise-induced vocal cord dysfunction. Laryngoscope. (2009) 119:1776–80. 10.1002/lary.20558 19572398

[33] Panchasara B Nelson C Niven R Ward S Hull JH. Lesson of the month: rowing-induced laryngeal obstruction: a novel cause of exertional dyspnoea: characterised by direct laryngoscopy. Thorax. (2015) 70:95–97. 10.1136/thoraxjnl-2014-205773 25260575

[34] Walsted ES Swanton LL van van Someren K Morris TE Furber M Backer V. Laryngoscopy during swimming: a novel diagnostic technique to characterize swimming-induced laryngeal obstruction. Laryngoscope. (2017) 127:2298–301. 10.1002/lary.26532 28236311

[35] Roksund OD Heimdal JH Clemm H Vollsaeter M Halvorsen T. Exercise inducible laryngeal obstruction: diagnostics and management. Paediatr Respir Rev. (2017) 21:86–94. 10.1016/j.prrv.2016.07.003 27492717

[36] Varney V Parnell H Evans J Cooke N Lloyd J Bolton J. The successful treatment of vocal cord dysfunction with low-dose amitriptyline - including literature review. J Asthma Allergy. (2009) 2:105–10. 10.2147/JAA.S6673 21437148 PMC3048598

[37] Doshi DR Weinberger MM. Long-term outcome of vocal cord dysfunction. Ann Allergy Asthma Immunol. (2006) 96:794–9. 10.1016/S1081-1206(10)61341-5 16802766

[38] Maat RC Hilland M Røksund OD Halvorsen T Olofsson J Aarstad HJ. Exercise-induced laryngeal obstruction: natural history and effect of surgical treatment. Eur Arch Otorhinolaryngol. (2011) 268:1485–92. 10.1007/s00405-011-1656-1 21643933 PMC3166603

[39] Maat RC Roksund OD Olofsson J Halvorsen T Skadberg BT Heimdal JH. Surgical treatment of exercise-induced laryngeal dysfunction. Eur Arch Otorhinolaryngol. (2007) 264:401–7. 10.1007/s00405-006-0216-6 17203312

[40] Bent JP III Miller DA Kim JW Bauman NM Wilson JS Smith RJ. Pediatric exercise-induced laryngomalacia. Ann Otol Rhinol Laryngol. (1996) 105:169–75. 10.1177/000348949610500301 8615579

[41] Norlander K Johansson H Jansson C Nordvall L Nordang L. Surgical treatment is effective in severe cases of exercise-induced laryngeal obstruction: a follow-up study. Acta Otolaryngol. (2015) 135:1152–9. 10.3109/00016489.2015.1062548 26200564

[42] Mehlum CS Walsted ES Godballe C Backer V. Supraglottoplasty as treatment of exercise induced laryngeal obstruction (EILO). Eur Arch Otorhinolaryngol. (2016) 273:945–51. 10.1007/s00405-015-3823-2 26541712

[43] Altman KW Mirza N Ruiz C Sataloff RT. Paradoxical vocal fold motion: presentation and treatment options. J Voice. (2000) 14:99–103. 10.1016/S0892-1997(00)80099-5 10764121

[44] Montojo J Gonzalez R Hernandez E Zafra M Plaza G. Office-based laryngeal injection of botulinum toxin for paradoxical vocal fold motion in a child. Int J Pediatr Otorhinolaryngol. (2015) 79:1161–3. 10.1016/j.ijporl.2015.04.044 26032616

[45] Goldstein R Bright J Jones SM Niven RM. Severe vocal cord dysfunction resistant to all current therapeutic interventions. Respir Med. (2007) 101:857–8. 10.1016/j.rmed.2006.08.009 17029770

[46] Christopher KL Wood RP Eckert RC Blager FB Raney RA Souhrada JF. Vocal-cord dysfunction presenting as asthma. N Engl J Med. (1983) 308:1566–70. 10.1056/NEJM198306303082605 6406891

[47] Marcinow AM Thompson J Chiang T Forrest LA deSilva BW. Paradoxical vocal fold motion disorder in the elite athlete: experience at a large division I university. Laryngoscope. (2014) 124:1425–30. 10.1002/lary.24486 24166723

[48] Mathers-Schmidt BA Brilla LR. Inspiratory muscle training in exercise-induced paradoxical vocal fold motion. J Voice. (2005) 19:635–44. 10.1016/j.jvoice.2005.03.005 16112543

[49] Johnston KL Bradford H Hodges HL Moore CM Nauman E Olin JT. The Olin EILOBI breathing techniques: description and initial case series of novel respiratory retraining strategies for athletes with exercise-induced laryngeal obstruction. J Voice. (2018) 32:698–704. 10.1016/j.jvoice.2017.08.020 29050661

[50] Shaffer M Litts JK Nauman E Haines J. Speech-language pathology as a primary treatment for exercise-induced laryngeal obstruction. Immunol Allergy Clin North Am. (2018) 38:293–302. 10.1016/j.iac.2018.01.003 29631737

[51] Bastian RW Nagorsky MJ. Laryngeal image biofeedback. Laryngoscope. (1987) 97:1346–9. 10.1288/00005537-198711000-00019 3669846

